# Encrusted cystitis caused by corynebacterium urealyticum: a case report with novel treatment strategy of intravesical dimethyl sulfoxide

**DOI:** 10.1590/S1677-5538.IBJU.2017.0588

**Published:** 2018

**Authors:** Tayyar Alp Ozkan, Mustafa Savas Yalcin, Ozdal Dillioglugil, Ibrahim Cevik

**Affiliations:** 1 Department of Urology, Kocaeli Derince Traning and Research Hospital, Kocaeli, Tukey; 2 Department of Urology, Kocaeli University, School of Medicine, Kocaeli, Tukey; 3 Department of Urology, Okan University, School of Medicine, Istanbul, Tukey

**Keywords:** Corynebacterium, Cystitis, Dimethyl Sulfoxide

## Abstract

Encrusted cystitis (EC) was first described as chronic cystitis with mucosal calcification in 1914 (1). It is a very rare chronic inflammatory disease presenting with dysuria, pelvic pain and gross hematuria. Voided urine contains mucus or calcified mucopurulent stone like particles. Urinalysis always reveals alkaline pH. It may be present in healthy individuals with no predisposing etiological factors (2-4). Etiologically, previous urological diseases, immunosuppression, urinary infection with urea splitting bacteria, or urological interventions resulting in bladder mucosa trauma may also be present (5, 6). In the present case report, we describe a novel treatment for EC with intravesical dimethyl sulfoxide.

## CASE REPORT

**Figures 1A and B:**
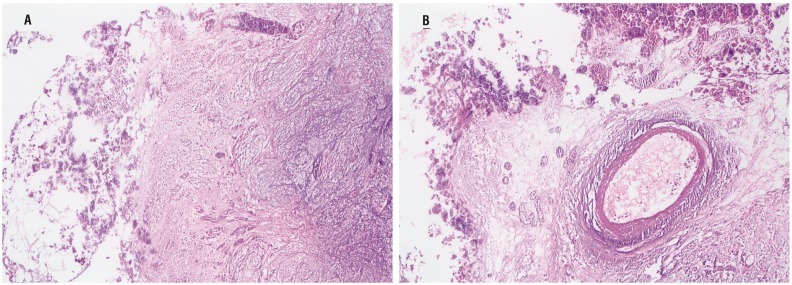
Bladder transurethral biopsy of encrusted areas 200xHE The deposits of calcium on the mucosa and chronic inflammatory infiltrate in the lamina propria.

We report a case of a 40-years old female with a history of transurethral bladder tumor resection (TUR-BT) in 2012. She was admitted to an outpatient clinic elsewhere in January 2015 with pelvic pain and recurrent urinary tract infection for the past 4 years. Diagnostic cystoscopy elsewhere revealed stone like particles covering the bladder mucosa. Later on, several TUR-BTs had been performed to remove these lesions in various hospitals elsewhere and histopathology reports revealed non-specific chronic cystitis without tumor. Patient had a re-TUR-BT in March 2015 again elsewhere for suspicious tumor, macroscopic hematuria and voiding stone like particles in urine. A necrotic bladder mucosa containing calcified encrustations with underlying inflammatory polymorphonuclear cell infiltration with abundant blood vessels was observed. Pathology result for this TUR-BT was encrusted cystitis (EC) (Figures 1A and B). Patient had a negative urinary tuberculosis screening, negative tuberculosis culture and PCR.

**Figure 2:**
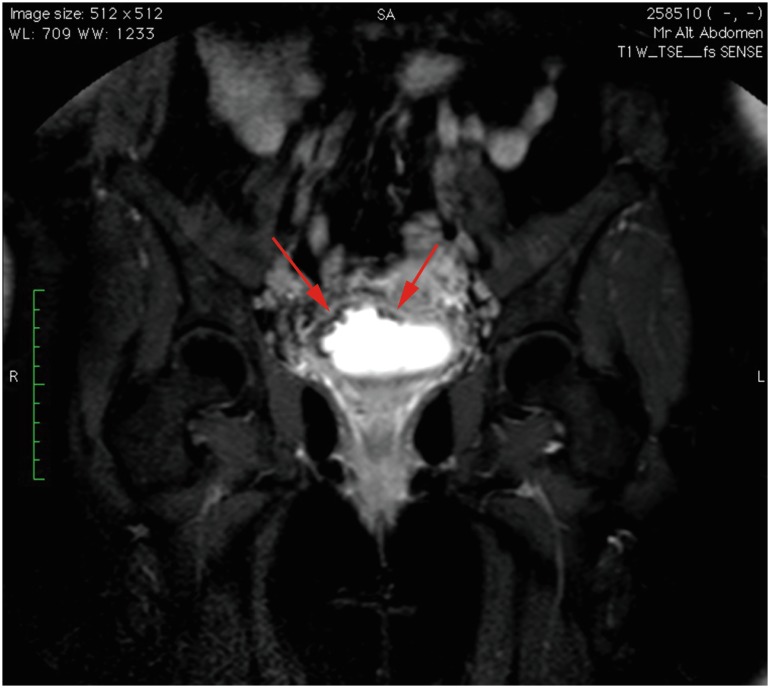
Coronal magnetic resonance image of bladder demonstrating filling defects (arrows) of right bladder wall.

Patient was admitted to our clinic with severe pain, gross hematuria, and voiding stone like particles, and she had lower urinary tract symptoms (LUTS) reminiscent of interstitial cystitis. Urinalysis showed alkaline urine with struvite crystals in the sediment. Her functional bladder capacity was 75 mL. Urine culture was sterile. An irregularity on the right bladder wall, and a moderate hydro-nephrosis at the right side was revealed with ultrasound. Filling defect at the dome and right bladder wall was also seen in magnetic resonance study (Figure-2). A diagnostic cystoscopy showed a calcified, hyperemic, fragile, edematous mucosa involving the whole bladder dome and right lateral wall covering right ureteral orifice. These lesions were completely removed with TUR, and part of the material and urine from bladder barbotage was sent for specific bacteriologic culture for Corynebacterium urealyticum, which was positive. Treatment was instituted according to antimicrobial susceptibility tests. For 2 weeks intravenous teicoplanin 400 mg/day (minimum inhibitory concentration 90% 0.5 micrograms/ mL), was given, and weekly intravesical dimethylsulfoxide (DMSO) treatment was started for 6 weeks. A standard solution of 50 mL of 50% DMSO (Rimso-50®) in aqueous solution (each mL solution contains 0.54 gr of DMSO) was administrated intravesically with a 10 French catheter, weekly for 6 weeks. Patient was allowed to void after 1 hour. LUTS relieved immediately. She had a cystoscopy with normal signs of bladder at 6^th^ month of follow-up. At 18^th^ month follow-up she was free from any complaint and infection with a remarkably increased functional bladder capacity of 340 mL.

## DISCUSSION AND FUTURE PERSPECTIVES

Urea splitting bacteria play a role in the EC etiology. A bacterial culture is essential for the bacterial confirmation of the condition. Though sterile urine culture should not rule out the diagnosis of EC, prolonged culture or tissue cultures should be performed. A prolonged 48 to 90 hours 5% carbon dioxide or sheep blood agar urine culture may be helpful when EC is suspected in the presence of sterile pyuria (3, 7).

Risk factors such as intravesical chemotherapy or BCG installations, urinary trauma, or bacterial urease activity results in ammonia release, which in turn damages the glycosaminoglycan layer of the bladder mucosa (2). As a consequence of this, characteristic encrustations composed of calcified plaques in the interstitium of the bladder mucosa occur.

Having a positive urine culture for C urealyticum does not necessarily means that patient has calcified encrusted particles in the urothelial mucosa. EC incidence was reported as 15.6% (n=18/115) for patients with positive C urealyticum urine culture (8). In this study, patients were treated with bladder instillation, and/or surgery in combination with antibiotics. The bladder had been irrigated with acidifying solutions such as, G-suby, Thomas, and N-acetylcysteine + acetohydroxamic acid + aztreonam combination. They reported that acidifying with these solutions was not completely effective to calcified particles. Alkaline urine is a product of a urea splitting bacteria. In our experience, we use DMSO in combination with TUR-B and antibiotics to resolve symptoms related to EC, though promising results were obtained even with a relatively high pH solution.

In our present case, the patient had a TUR-BT history, and she presented with LUTS, gross hematuria and micturition of sandy grits. Urine culture was sterile, and ultrasound revealed stone like particles. Patient had a re-TUR-BT and part of the material and urine from bladder barbotage was sent for C urealyticum culture, which was positive. Treatment with teicoplanin 400 mg/day, and weekly intravesical DMSO was started.

C urealyticum is the most commonly reported cause of EC, and it has multiple antibiotic resistance (9). Teicoplanin, vancomycin and glycopeptides are the antibiotics of choice in the first line treatment of C urealyticum. However, EC usually does not respond to antibiotic treatment alone. The bacteria contained in calcified plaques prevent antibiotic penetration. Multimodal treatment consists of plaque resection, urinary acidification and antibiotic treatment (3).

To the best of our knowledge, DMSO has never been reported as treatment agent for encrusted cystitis. DMSO does not act as an acid or base with a pKa=35. It is an important polar aprotic solvent that dissolves both polar and non-polar compounds. It is miscible in a wide range of organic solvents as well as water. DMSO is a FDA approved drug mainly used in interstitial cystitis/painful bladder syndrome. DMSO plays a role in replenishing the damaged glycosaminoglycan layer and it has anti-inflammatory activity on injured bladder urothelium (10). The presence of osteocalcin and ostonectin in EC urothelium has hypothesized that EC may be related with systemic inflammation (11). DMSO is a weak acid with pH of 6.7. It is an aprotic solvent that replenish glycosaminoglycan layer and has anti-inflammatory effect on urothelium. We can hypothesize that anti-inflammatory treatment enhancing urothelial recovery may improve patient's symptoms and treat EC. In our patient, no discomfort except sensation of bladder burning was observed. Quite contrary to acidic solutions, pain and swelling due to DMSO treatment were caused by bladder inflammation or irritation.

In conclusion, rare pathologies such as EC are limited to case reports and case series. Although urine acidification has been reported to be a component of the treatment, bladder irrigation with an aprotic solvent was effective in our case, and has not been reported previously. Further studies are needed to confirm our treatment effect and results.
